# Christian Science Methods

**Published:** 1899-08

**Authors:** Nelson W. Wilson

**Affiliations:** Buffalo, N. Y.


BUFFALO MEDICAL JOURNAL.

VOL. XXXIX. AUGUST, 1899. NO. 1.

### Original Communications.

### CHRISTIAN SCIENCE METHODS.

By NELSON W. WILSON M. D., Buffalo, N. Y.

THE christian science evil has become so prominent of late by reason of the unusual number of deaths which have followed the administration of prayer, that the authorities in almost every big city in this country have been forced to take some action looking to a checking of the system of treatment.

In Buffalo, the recent Saunders case at Fort Porter, while not the first death from neglect, was the most prominent because of the general surroundings; and further, because it happened within the jurisdiction of the United States authorities. There is also another fact which will add interest. This is that it was by the merest chance that the christian scientists were thwarted in their efforts to hush up the matter and hurry the body under ground.

If there is one kind of man who knows more about things in general than any other kind of man it is the United States soldier—the seasoned private or non-commissioned officer. He has grown into the habit of keeping his eyes and ears open and his mouth shut. When he talks he is invariably interesting. When he is serious and you know him, you can depend upon him. It was thus that I got to be fairly well acquainted with christian science and christian science ways, while serving as assistant-surgeon at Fort Porter last fall and winter. The soldiers knew much of christian science; some of them had read Mary Baker Eddy's book and the magazine published in the interest of the organisation and were well versed in the ground work of the faith. Others—one in particular—had taken christian science treatment, and had returned to the hospital office for relief from a trouble, which Neisser discovered was due to a gonococcus. From these men I gathered much interesting information, and in speaking of the death which must necessarily follow neglect, in serious cases, my informant, who was a particularly intelligent man, said:

> The reason that so little is said, is because whenever they see a person is going to die, the "healers" abandon their work, and the family sends for a strange doctor. They tell him how sudden was the illness and all that sort of thing and when the patient dies they get a death certificate and everything is smoothed over.

There are reasons why this man's opinion was valuable,—reasons which cannot here be stated. Suffice it to say, he had studied christian science, was hand and glove with the scientists for a long time and had taken the "treatment." He is not now a believer, and I presume should he meet the soldiers' fate in the far-away Philippines, it would be credited to his backsliding and not to the penetrating powers of a Fillipino bullet.

I had wondered often how the scientists secured permission to bury their dead. The explanation of the soldier solved the mystery, and at the same time showed the great laxity in furnishing certificates on the part of physicians.

When the Saunders boy was first taken ill at Fort Porter, there was the general post gossip concerning his illness,—such as naturally follows the illness of any child; a little more maybe, in view of the fact that he was the child and relative of christian scientists. He was living at a government post. Dr. Walter D. McCaw, captain and assistant-surgeon U. S. Army, was in charge, and I was his assistant. At no time was Fort Porter without the services of a medical officer, who attended all cases at the post without fee. The Saunders boy, as has been told, went from bad to worse, and finally died. As his breath was leaving his body, Dr. McCaw was hurriedly sent for. Very properly he refused to give a certificate and reported the matter to the coroner's office, where, immediately a mystery was made of it. It was kept from the newspapers,—by what influence has not yet appeared. The greatest secrecy was maintained in all quarters. At the Fort that morning I met one of the post mortem examiners, who had been sent to view the body and give a certificate,—in fact, he was *instructed* to give a certificate. I suggested that the death occurred on government property, that a physician was within call at all times, that one had been sent for at the time of death, and had refused to give a certificate, and that an investigation was within the range of possibility. Under these circumstances, and in view, too, of the fact that there was scarlet fever at the post, I thought he would be remiss if he did not determine the exact cause of death before giving a certificate.

My suggestions were acted upon, the case was reported to the District Attorney of Erie County, thence to the U. S. District Attorney, and post mortem disclosed the cause of death to have been pneumonia.

Had the officials gone on as they were first headed the secrecy of the coroner's office would have been maintained, the child would have been buried quietly, and christian science would have said he "passed on" owing to some evil influence.

Since that time the newspapers have been full of christian science "passings on," the most horrifying of all the cases happening at a little place called Bowling Green, near Toledo, O. There a four-year child died in great agony from the presence in his throat of a grain of corn. A local physician informed the family that an operation was urgently required, but the family turned over the case to a female faith curist.

In explanation of the child's death this woman says there was a lack of faith on the part of the boy's father, which interfered with her ministrations.

In Chicago, New York, Brooklyn, Philadelphia and even Boston, the head center of the cult, there have been cases almost as inhuman.

Just what christian science is and how it is taught has been ably exploited by a reporter of the New York *World*, who became a healer for the purpose of giving to the public the truth. He spent three weeks studying in an institute at Lansingburg, N. Y., paid $100 and got a certificate.

In his report he tells many strange things hitherto unknown to the public. His introduction is racy reading, too. He says:

Empowered as I am, I have the right to go into a sick room and banish from it the physician and nurse who may be in attendance there. I can forbid their entrance into the room after my coming upon the scene. I can take all the medicine bottles, as I should do if I am a good straight christian scientist, and throw them out of the window. If my patient is swathed in bandages I can take them off or tear them off, as sometimes I might have to do, and leave gaping wounds exposed to the naked air and the healing influence of christian science.

Should my patient be suffering from a dozen different fractures of bones or strains of tendons, I can remove all the splints and feel assured that the broken ends of the bones will be held together by the simple influence of mind.

To go still further, I can let the little sick, suffering child, who is battling with fever struggle for breath unaided by anything to relieve it but the supposed irresistible influence of love. As its life ebbs away, breath by breath, I can still refuse to allow any physician to come near the room while I sternly maintain that nothing is needed there but myself and the tenets and principles of Mrs. Eddy's Science and Health.

If the helpless cripple with spinal trouble is brought to me, whose poor frame is supported by the latest and most improved system of steel frame work, I can at once taboo all such paraphernalia and say: "This wretched invalid needs nothing of the kind. His limp backbone needs but the strength of mind. His flaccid joints require nothing but recognition of the fact that there is nothing really the matter with him. If he wishes and I wish and the Divine Principle wishes, he can walk in any go-as-you-please contest to-morrow."

All of this I can do, so say the christian scientists, because I have taken my regular course of study. I own the degree of "C. S." and I am thoroughly qualified to banish sickness from that part of the world which I can reach.

The only exception to this treatment, which is admitted by christian scientists is in cases of obstetrics. There the christian scientist bows in humility and submission to the accoucheur and the midwife. As a body they go even further. They have schools of obstetrics and midwifery of their own, and while still adhering to the infinite influence of mind and the spirit, they still, strange to say, admit and practice all that is known to the regular practitioners of the most orthodox school of medicine.

One startling truth brought out by this course of study was that christian scientists seldom or never practice for love, or do charitable work, even among the poor. Later this will be referred to more specifically.

When the novice first went to Lansingburg, he saw Mrs. Harriet L. Betts, the chief healer and "first-reader," who was extremely suspicious and cross-questioned him severely, as to whence he came, who sent him and whether he had ever been healed by treatment. She was very reserved, and held out little hope. Finally, she relented and agreed to take the reporter as a student after very rigid questioning. The writer says:

Mrs. Betts did not ask me if I was a reporter, but said that she was afraid at first that I was.

"You know," she said, "that in these days of persecution we have to be very, very careful. The newspapers have been trying all they can to find what they can say against christian scientists."

"Is there anything christian scientists have to hide?" I asked.

"No," she said boldly, "there is not. But our cures have been so marvelous and so many that we have the whole medical fraternity against us. Not only that, the Roman Catholic Church has determined to join in with the medical men and fight us tooth and nail."

Before the reporter was finally admitted to the class he had to subscribe to these questions:

1. Do you still believe in drugs or medicines of any sort?
2. If you were taken sick yourself would you send for the doctor?
3. Would you place yourself in the hands of a christian science healer?
4. Are you convinced that God is all in all, and that mind is all that is needed to correct error?
5. Are you sincerely in earnest in wishing to become a christian science healer, and do you agree faithfully to adhere to the tenets of the mother church as laid down in the church manual?
6. Do you recognise that the writings of Mrs. Dr. Mary Baker Eddy are the only authoritative works in christian science, and do you look upon Mrs. Eddy's Science and Health, with Key to the Scriptures, as our principal text-book?

Naturally, every lesson in christian science requires a book, and this book is written by Mrs. Eddy. The books average $5.00 each, and the quality averages up to about a 50 cent volume in a bargain-day sale. There are $3.00 volumes, but you must not get them—they are too bulky. The $5.00 books are the ones recommended.

The reporter's first lesson was a plunge into the "recapitulation," and of this first instruction he writes:

Some parts it was indispensable that I should memorise word for word. To begin with:

Question—What is God?

Answer—God is divine principle, supreme incorporeal being, mind, spirit, soul, life, truth, love.

Question—What is the scientific state of being?

Answer—There is no life, truth, intelligence nor substance in matter. All is infinite mind and its infinite manifestation, for God is all in all. Spirit is immortal truth; matter is mortal error. Spirit is the real and eternal; matter is the unreal and temporal. Spirit is God, and man is his image and likeness; hence, man is spiritual and not material.

Those two questions and answers form the basis of christian science teaching. They are also a trap for the unwary student, for at any moment the teacher will single out some individual member of the class and ask him, "What is God?" If he makes one mistake in phraseology he will receive a black mark in the books.

The second question sprung upon you unawares, never fails to confuse. To prove this let any one take these two questions and answers, memorise them today with all possible care and see how nearly he can write them out a week hence with close attention to punctuation and capitalisation.

He will then be able to realise one of the first stumbling blocks in the study of christian science.

When I had promised that I faithfully would study these questions and answers over night my first lesson began. It consisted of a series of statements on the part of the teacher, backed up liberally from quotations from "Science and Health" and the bible, to show that there is nothing in the universe but Mind. That Mind is God and that man is but the reflection of God and, therefore, the reflection of mind.

In order to begin the study of christian science with any degree of understanding several things must be taken for granted. Christian science is about 1,000 times more unintelligible to the beginner than the first book of Euclid is to a dull schoolboy.

As in Euclid, christian science contains several so-called axioms from which you are never allowed to depart. The first one is that God is All in All, that God is Mind, that God is good; that, therefore, if God is All in All, and there is nothing but God or Mind, and if God is good, there can be nothing which is not good.

So it followed that so-called sickness does not really exist, because it is not good. Sin also has no existence, as that is evil. As God is all-powerful he will not permit the existence of sin, because he will not allow anything that is not good.

This is difficult for the ordinary, common-sense business man to understand. If you say you do not understand it the scientist says it is because you are not able; that he can understand it thoroughly, because he has reached that stage of his progress which he calls "realisation."

The teacher tried to make it easier for the new students by various similes. For instance, there is no such thing as sickness, she said, because God is good and all-powerful. The imaginary ailments of mortal men simply seem real to the sufferer because he does not realise the actual presence of God and Mind.

The blind man cannot see the light because his eyes are closed to it. The light is there, nevertheless. A dark room really is not dark, because there is no such thing as darkness. It simply appears dark because the light is not there, and in any case it is impossible to get a room so dark that there is not some degree of light.

In the same way there is no such thing as cold. It simply is the absence of heat. To prove this there is no known degree of cold obtained, but a still colder degree can be reached. In other words, there is no negative to anything. All is positive.

In further proof the teacher went on to show that silence is the absence of sound, cold the lack of heat, darkness the want of light, sickness and sin the need or non-realisation of Divine Mind.

When this had been drilled in the student for two hours, he was obliged in self-defense to contradict it or admit it was so. An admission that he did not comprehend it endangered his position in the class. It was much better boldly to deny these things than to say "I do not understand."

In the latter case he was looked upon as hopeless and little attention was paid to him. In this first lesson no attention was paid to healing. It simply was a sort of groundwork laid as a foundation for the supposed state of perfection necessary in order to become a healer.

When students are pronounced perfect in the recapitulation they are taken into the healing mysteries. They are called "perfect"

when they can answer these questions satisfactorily according to Science and Health.

What are spirits and souls?
What is the science of soul?
What is substance?
What is life?
What is intelligence?
What is mind?
Are doctrines and creeds a benefit to man?
What is error?
Is there no sin?
What is man?
What are body and soul?
Do not brains think and nerves feel, and is there no intelligence in matter?
Is it important to understand these explanations in order to heal the sick?
Does christian science or metaphysical healing include medication, hygiene mesmerism or mediumship?
Is not materiality the concomitant of spirituality, and is not material sense a necessary preliminary to the understanding and expression of spirit?
You speak of belief. Who or what is it that believes?
Do the five corporeal senses constitute man?
Will you explain sickness and show how it is to be healed?
How can I progress most rapidly in the understanding of christian science?
Have christian scientists any religious creed?

Now comes some startling revelations. Says the reporter:

I was surprised to find that christian scientists do not ignore diagnosis. They do their best to determine the exact disease in every case they treat. This they do by symptoms, the same as the ordinary physician.

In the next important step, however, they inquire into the cause or "causation," as they call it. In this they differ radically from the regular practitioner. All diseases are looked upon as "claims" or "beliefs."

The patient is said to suffer from a "belief" of a bald head or a "claim" of cancer. His head is not really bald—he simply cannot see the hair there, or part it either, for that matter. Another invalid would be said to have a "claim" of carbuncle. The carbuncle is not there—it does not exist. He simply is foolishly poulticing his evil imagination. In what might be termed the pathology of christian science diseases are classified somewhat roughly. The cause of rheumatism, for instance, is not exposure to cold and wet, but exposure to the hatred of enemies.

No amount of water in the trenches, according to christian science, could ever have given rheumatism or fever to an American soldier at Santiago. But a malicious thought on the part of his Spanish enemy would be liable to throw him into the hospital for months.

The teacher gave an instance of rheumatic fever in which she frankly admitted she had erred in treatment. By a diligent inquiry among the man's neighbors she thought she discovered that her patient had a violent hatred for his mother-in-law. She proceeded with her treatment on those lines.

She brought herself to realise that this poor man could not hate his mother-in-law. It was impossible. She knew that God made mothers-in-law, just as He made everything else. All that God made was good. Therefore, mothers-in-law must be good, no matter what people might say to the contrary.

Strange to say, the man's rheumatism did not get better, and the teacher was worried. It was not until she found out that the man had no mother-in-law, never having been married, that she was able to bring her treatment in a direction to cure him.

From this on the lessons were full of these anecdotes, or "demonstrations," as the teachers called them, and became highly interesting.

All accidents resulting in sprains, or burns, or broken limbs are caused by fear, not necessarily on the part of the unfortunate who met with the accident, but in the mortal mind, perhaps of some one near and dear to the sufferer. In consequence of this it is very important to conquer all semblance of fear whenever it is met. No mother could safely bring up a child if she was afraid something was going to happen to it. It was only by realisation that the child was safe from all harm that it could be prevented from falling down a well or getting run over by a trolley car.

Now here is something which will interest Dr. Park. For years and years the medical profession has been working and studying to determine the source of cancer, and now the christian scientists dismiss it in a breath, saying it is caused by jealousy. All Dr. Park has to do now is to stamp out jealousy and he will have the cure for cancer.

Liver troubles are caused by interfering with other people's business, and neuralgia is the result of covert sneers and ridicule. Now let the story be told by the reporter:

But before teaching us anything in the way of practical healing the principal asked us in a body what we thought ourselves would be the proper method of procedure.

"Now we will suppose," she said, "that you are called into a sick room where there is a little child suffering with a 'claim' of whooping cough. What is the first thing you would do?"

There were various replies to this. One student said that he would leave word at the door that the doctor should not be allowed to enter the house.

"Good," said the teacher, "but there is something far more important than this."

Another student suggested that she would open her "Science and Health" and look the case up in that text-book.

"That is right, too," assented Mrs Betts, "but you seem all to have forgotten the main thing. Therefore, I will take an imaginary case of whooping cough and tell you first how you should go to work and treat it.

"In the first place half the battle ought to be won before you reach the house. When you are summoned, you will probably be told the nature of the case. You will hear that the child is very low and perhaps not expected to live. The first duty of the true, earnest christian scientist is to treat him or herself before he ever reaches his patient. When you are told that the child has whooping cough you will at once say to yourself, 'This is not so; there is no such thing as whooping cough. God made that little child the same as he made us all and the Divine Mind certainly never intended that the poor little frame of that chiid should be racked by anything so distressing as whooping cough.' If by the time you reach the sickroom you have been imbued with this thought, with a proper sense of 'realisation,' you will find that there is already a change for the better.

"If the anxious parent has been putting goose oil on the child's throat you will tell her to throw the bottle away. If the little chest of the baby is wrapped up in flannels you will take them off. You must then insist that no one enter the room but the mother, and not even she while you are there.

"Now comes the hardest part of your work. There never was a case of whooping cough or measles or scarlet fever but what was caused entirely by maternal fear. Every fond mother who starts in to raise a child waits anxiously for the period when her offspring is mortally supposed to be subjected to the various children's diseases. Where that fear exists to any extent there is no possible escape for the child, consequently you will, in a case of this sort, help to treat both mother and child.

"By constant, earnest realisation you must remove all fear from this mother's heart. Then you will see how much you are helped in treating the child itself. Never for one moment must you forget to go back to the first principle of christian science. You must remember that God is all in all; that sickness is but an error; that error does not exist in reality, being but the absence of truth.

"Now," said the teacher, with a beaming smile, "don't you see how easy it all is?"

To some of the class it must have appeared perfectly easy, for there were loud vociferations of assent.

"When once you have restored the truth to that poor little suffering child error will have departed, and if it be a new case to you you may be just as surprised at the sudden recovery as the troubled parents are. Let me tell you right here that the most perfect cures of whooping cough have been accomplished instantly, or in less than five minutes. In the many dozen cases that I have treated myself a change in the child's breathing has been noticed almost as soon as I entered the room."

"But supposing," ventured one student, who had not the faith of some of us, "that the breathing of the sufferer does not improve? Supposing the baby does not seem to get well and looks as though it were going to die?"

"Babies never die," corrected the teacher. "But if you should enter the sick room with any such fear as you now express it is quite possible that the child would 'pass on' or—as the mortal mind calls it, 'to die.' "

"But," persisted the doubtful student, "supposing I had no such fear when I entered the room. Supposing I fully realised that there can be no such thing as error, that mind is superior to anything, and the child does not improve, but seems to get into a more dangerous condition; is not that possible, even though my own realisation of the truth be perfect?"

"Yes," assented Mrs. Betts, "that is possible and sometimes happens, but such a contingency is always caused by one thing. When you find that in spite of all your efforts you have made little or no progress you will find that somewhere in that household there is both scepticism and malice. Somebody in that family has been opposed to your coming there. In that case it will be your duty to find out who it is. In this it will be necessary to exercise a certain amount of tact.

"When you find out your opponent, generally speaking, it will be the father and not the mother, or perhaps one of the grandparents, you must devote your attention to him. You must take him to one side and bring to bear upon him all the tact which you can summon to your aid. It will be better to appeal to him first in a material way. Ask him if it is not a fact that before you came the doctor had not done any good, anyway. Ask him if he doesn't know that physicians kill ten times as many persons as they cure.

"Put it to him, even if he does not believe in christian science, whether he does not consider that medicine and drugs in ninety-nine cases out of a hundred do more harm than good. Then when you have got him to admit all this leave it to his sense of justice whether he is not willing that the child's life should be saved, even though it be accomplished by something he does not understand or believe in.

" At the same time you must never forget that there is no real opposition either to you or to divine science in this man's heart. It is simply that the truth is not there. I have come in contact with many cases of this sort and never failed with the proper amount of tact and earnest realisation to win a victory for the blessed truth."

The particular instruction given in whooping cough coincides with the treatment of all children's diseases, and christian scientists, from my personal observation, seem to have a large number of such ailments placed in their hands.

The next case taken up by the teacher was that of pneumonia, and I listened anxiously to this treatment, as I had been unfortunate enough to suffer with that generally fatal trouble myself.

It is insisted by christian scientists that they have never lost a case of pneumonia. They point to the large list of deaths from this disease where nothing but medical treatment has been used. The teacher then went on to give the history of a case of inflammation of the lungs where she had encountered any amount of family opposition.

The wife of the man who suffered was a christian scientist, but the mother of the sick man was an avowed enemy of anything of the sort. It was a battle between wife and mother-in-law. The doctor who had been treating the case had left a Spanish fly blister to be applied. This the wife's mother-in-law was determined should be used according to the doctor's directions.

The sick man himself was too near death, apparently, to object to any sort of treatment.

In the difficulty that confronted the science doctor under these circumstances she made up her mind that the Spanish fly blister could do no harm in any case. She brought herself to realise that Spanish fly was an error; that on a true child of God it would be powerless to operate.

"And," she continued, her face shining with triumph, "although the Spanish fly was left on the man's chest for over twenty-four hours, it failed to raise a blister! There was scarcely even a red spot where it had been applied."

From the whole course of my study it appears to me that the basis of treatment in all cases of disease by christian science is the same. The only difference is in the careful selection of "causation," or the alleged original cause of the disease.

Rheumatism is caused by hatred. In its treatment it is necessary to discover where the hatred exists, and then by realisation that God and mind never intended that there should be any hatred in his dominion, that in fact there is no hatred in existence, but only the imagination of it—in other words, the error of it, or the corresponding absence of love—to bring about a reassertion of the truth.

With the eighth lesson of our course the students were supposed to be well on in practical treatment or healing.

Although each separate disease is taken in detail, the treatment in itself was always the same thing, simply a realisation on the part of a healer to overcome the error that appeared to exist by the reassertion of truth.

But before leaving practical treatment and healing, our attention was called to several difficulties which were bound to present themselves to young beginners in the labor of Christian science.

During the whole time of our tuition we students had not only faithfully to attend to class but we were forced constantly to study our text-book—Science and Health. We did this. There was no escape from it. We were at all times liable to individual examination as to the progress we had made in that respect.

Now that we were supposed to be sufficiently advanced in the pathology and therapeutics, as you might call them—and as christian scientists do call them—of christian science, our attention was brought to bear upon the obstacles that would present themselves to us in our practice. The first and most dangerous of these was what they called "chemicalisation."

Chemicalisation is described in Science and Health as: "The process which mortal mind and body undergo in the change of belief from a material and spiritual basis. The upheaval produced when immortal truth is destroying erroneous mortal belief. Mental chemicalisation brings sin and sickness to the surface, as in a fermenting fluid, allowing impurities to pass away."

This christian scientists attempt to explain by showing what happens when an acid and an alkali meet and ferment, bringing out a third condition, or salt. Chemicalisation often aggravates the symptoms of disease, they say, and will generally account for a patient getting worse instead of better.

Many of the students, and I know I did, hailed this new development with delight. It seemed as though it would be a handy thing to account for apparent failure.

It was good to know that no matter how strong might be your own individual realisation, if your sick man obstinately refused to improve or even seemed to get worse the trouble could be accounted for by this welcome term, "chemicalization."

If, for instance, you were treating a case of typhoid fever with no effect whatever and if much to your consternation the patient developed new troubles in the shape of boils, or anything else, it was simply "chemicalisation."

It was the truth combating with error for victory and coming out in the shape of an eruption. We were gravely informed by the teacher that, in some cases, "chemicalisation" might be displayed so severely that the patient would "pass on," in other words, die in spite of all our efforts.

Next to persecution "chemicalisation" is said to be the greatest enemy and drawback to christian science. From what I could gather "chemicalisation" was often caused by persecution, that is, the struggle between truth and error. But in spite of the fact that I paid close attention to everything that was taught me regarding "chemicalisation," I could get at no certain means of overcoming it.

The truth I was told was bound to prevail in the end, yet, it was frankly admitted that chemicalisation had occasionally caused death, or "passing on." We received the comforting assurance that as we became greater in our power of healing we would meet less of the bugbear, "chemicalisation," until it would finally disappear altogether.

The only conclusion I could come to with regard to the handling of this enemy was to follow an advice much resembling that of "Chuck" Connors, who tells you to "Forget it."

The next serious foe we were told we should have to meet was a sort of twin enemy—malice and envy. These enemies would appear to us mainly in the shape of regular physicians and clergymen.

The medical fraternity, christian scientists say, cannot help envying christian scientists, because of the wonderful cures the latter perform wherever they go. The malice of the clergy is encountered because christian science teaches people to think for themselves.

The first duty of a christian scientist is not only to banish the medical man from the sick room and throw away his medicine, but bring the patient to a condition where he has lost all faith whatever in any sort of drugs.

In this connection, however, we were taught that a certain amount of liberality was not always ill-advised. If a patient wore bandages that had been put on by a physician it was not always necessary to take them off.

While, as chrisian scientists, we felt convinced that bandages could do no good, still, with a proper realisation of the truth, we could prevent them from doing any harm, so that where their removal might provoke anger or malice it was sometimes a good thing to let the patient wear them.

The same thing applies to sticking-plaster. While plaster cannot possibly help a cut or a wound it cannot hinder what may be accomplished by mind. Consequently the liberal-minded christian scientist does not taboo sticking-plaster. But in all cases ointments and lotions are sternly forbidden, as they contain drugs.

A case which I saw treated myself of badly chapped hands was treated with nothing but love. The first command to the patient was that all the cold cream which he had applied should be at once washed off.

Baldness, scientists claim, has repeatedly been cured by christian science, where all other applications have failed.

To the skeptical student, and I must confess I was one, there were many glaring inconsistencies that I noticed even among the most devout christian scientists. On special occasions where the class listened to testimony from many patients who had been healed there were several cases given where decayed teeth had been replaced, simply by the influence of mind.

Yet I noticed that many of the scientists did not hesitate to have recourse to the practical work of the dentist. Gold fillings in christian scientists' teeth are quite conspicuous, as they are very careful of their personal appearance. If I should judge the rest of the class by my own feelings, I know that the last lesson was hailed with delight by all of us.

We were by this time enabled to talk glibly of mind, soul, spirit and love, and some of the class maintained stoutly that they had reached the stage known as "realisation." For my own part I

must frankly confess that I had not, yet the teacher asserted several times that I was the most promising student she had.

At the final lesson and graduation each individual member of the class was closely questioned. The student had supposititious cases of sickness given to him, and was asked what he would do.

It seemed that we fully came up to the standard, for Mrs. Betts informed us that she looked upon her May, '99, class as the most gratifying one she had had.

There was quite a lot of sentiment and affection in the address in which she bade us farewell and sent us out into the world with our degrees of "C. S." Then she touched upon a few matters that were certainly of great importance to those of the class who really intended to practise christian science healing.

One piece of advice was undoubtedly good and welcome to all of the students, except myself.

"When you begin to practise," she said, "you may, perhaps, have some scruples about accepting payment for your work. I want to impress upon you that you must banish all such silly ideas from your minds at once. You must not only make people pay you, but you must make them pay you well.

"Remember all the doctors' bills that you are going to save them; think of the enormous amount of money that these poor mortals pay out every year for drugs and medicines that cannot possibly do them any good. If it is right to pay a doctor for having no effect upon you whatever, surely it must be right to pay for positive help and relief. I want to warn you all against using your personality. No matter how attractive you may be personally, you must sink that and rely entirely upon mind. The moment you allow your personality to creep into your work you are approaching dangerously near hypnotism. There is nothing good in hypnotism. It was never intended by the divine mind that one person should have any personal power over another."

And that is christian science!

